# Data-driven, voxel-based analysis of brain PET images: Application of PCA and LASSO methods to visualize and quantify patterns of neurodegeneration

**DOI:** 10.1371/journal.pone.0206607

**Published:** 2018-11-05

**Authors:** Ivan S. Klyuzhin, Jessie F. Fu, Andy Hong, Matthew Sacheli, Nikolay Shenkov, Michele Matarazzo, Arman Rahmim, A. Jon Stoessl, Vesna Sossi

**Affiliations:** 1 Division of Neurology, Department of Medicine, University of British Columbia, Vancouver, British Columbia, Canada; 2 Department of Physics and Astronomy, University of British Columbia, Vancouver, British Columbia, Canada; 3 Pacific Parkinson’s Research Centre, University of British Columbia, Vancouver, British Columbia, Canada; 4 Department of Radiology, Johns Hopkins University, Baltimore, Maryland, United States of America; Banner Alzheimer’s Institute, UNITED STATES

## Abstract

Spatial patterns of radiotracer binding in positron emission tomography (PET) images may convey information related to the disease topology. However, this information is not captured by the standard PET image analysis that quantifies the mean radiotracer uptake within a region of interest (ROI). On the other hand, spatial analyses that use more advanced radiomic features may be difficult to interpret. Here we propose an alternative data-driven, voxel-based approach to spatial pattern analysis in brain PET, which can be easily interpreted. We apply principal component analysis (PCA) to identify voxel covariance patterns, and optimally combine several patterns using the least absolute shrinkage and selection operator (LASSO). The resulting models predict clinical disease metrics from raw voxel values, allowing for inclusion of clinical covariates. The analysis is performed on high-resolution PET images from healthy controls and subjects affected by Parkinson’s disease (PD), acquired with a pre-synaptic and a post-synaptic dopaminergic PET tracer. We demonstrate that PCA identifies robust and tracer-specific binding patterns in sub-cortical brain structures; the patterns evolve as a function of disease progression. Principal component LASSO (PC-LASSO) models of clinical disease metrics achieve higher predictive accuracy compared to the mean tracer binding ratio (BR) alone: the cross-validated test mean squared error of adjusted disease duration (motor impairment score) was 16.3 ± 0.17 years^2^ (9.7 ± 0.15) with mean BR, versus 14.4 ± 0.18 years^2^ (8.9 ± 0.16) with PC-LASSO. We interpret the best-performing PC-LASSO models in the spatial sense and discuss them with reference to the PD pathology and somatotopic organization of the striatum. PC-LASSO is thus shown to be a useful method to analyze clinically-relevant tracer binding patterns, and to construct interpretable, imaging-based predictive models of clinical metrics.

## Introduction

Pathological processes associated with neurological disorders often develop in distinct spatio-temporal patterns. These patterns can be imaged with positron emission tomography (PET) or single-photon emission computed tomography (SPECT) using appropriate radiotracers. However, traditional quantitative PET and SPECT image analysis metrics, such as the standardized uptake value (SUV) and non-displaceable binding potential (BP_ND_), are often evaluated as averages over a specific region of interest (ROI). This approach neglects the spatial distribution of radiotracer binding, which may be affected by disease within the ROIs. There is thus a growing realization that better methods of spatial image analysis are needed to achieve a more complete disease characterization and to improve prediction and tracking of disease progression [1–3]. The primary approach to spatial analysis in the context of these objectives has so far relied on the use of texture- and shape-based image features [4–8], leaning heavily on the emerging field of radiomics [9–11]. Following a general radiomics approach, a large number (between tens and hundreds) of different features are computed from the images within pre-defined ROIs. The features are then used as inputs to one of the established machine learning models (neural nets, decision forests, etc.) to predict clinical measures of interest [12–14].

However, investigations that use radiomic features are limited in two aspects. First, PET and SPECT brain imaging studies often have a relatively small sample size (10 to 50 subjects) compared to the number of tested features (“large *p*, small *n*” problem) [14–17]. The values of features may likewise depend on internal variables required to define the features, image reconstruction algorithm and ROI definition criteria [7, 18–21]. This aspect may result in a very large model-parameter search space and substantially increase the risk of model overfitting, particularly if the employed statistical testing strategy is not robust or valid [22–25]. Second, a relatively high complexity and locality of the radiomic features hinders visualization and biological interpretation of the disease-relevant spatial patterns [26].

In the present work we explore a data-driven approach to the analysis of PET brain images that aims to overcome the shortcomings described above, i.e. overfitting, optimal feature search, and interpretability. In contrast to relying on generic radiomic features, our method identifies disease- and structure-specific spatial patterns of radiotracer binding on a voxel level, and constructs image metrics optimized for prediction of clinical measures. The method uses individual-subject voxel values as inputs, and consists of two steps. In the first step, the high dimensionality of the voxel data is reduced in a blind (i.e. independent of the clinical metrics) manner using principal component analysis (PCA). In the second step, several principal components (PCs) with the highest percent of explained total variance are selected and linearly combined to predict clinical metrics of interest. The combinations are determined using regularized fitting by the least absolute shrinkage and selection operator (LASSO) [27]. In comparison to ordinary multivariate fitting, LASSO selects only those input variables that improve prediction accuracy; inputs that do not contribute to the accuracy are set to zero. We refer to this method as the principal component LASSO (PC-LASSO), and to the best of our knowledge, it has not been previously used in the analysis of PET images. The use of sequential dimensionality reduction and variable selection techniques, wherein PCA is blind and LASSO is used in conjunction with model cross-validation, reduces the risk of overfitting, and enables the use of raw data from 10^3^–10^4^ voxels to build predictive models from a much lower number (10–100) of subjects. The fitted PC-LASSO models can be used to compute PC-LASSO estimators, which capture information in a non-local manner by assigning a weight to each voxel that quantifies the voxel’s relevance to the predicted clinical metric. The weights can be visualized in the context of the topology of the imaged structure, providing direct information on the spatial characteristics and evolution of disease.

We test the PC-LASSO method by applying it to high-resolution PET images of subjects suffering from Parkinson’s disease (PD), the second most common progressive neurodegenerative disorder after Alzheimer’s disease [28]. PD is associated with degeneration of pre-synaptic dopaminergic terminals in the basal ganglia, while initially inducing a compensatory response in the postsynaptic neurons [29, 30]. Accordingly, the method is applied to striatal PET images obtained with a pre-synaptic and a post-synaptic dopaminergic tracer: ^11^C–dihydrotetrabenazine (DTBZ), a marker of the vesicular monoamine transporter type 2 (VMAT2) that reflects the density of dopaminergic terminals, and ^11^C–raclopride (RAC), a dopamine D2 receptor antagonist. We tested whether PC-LASSO can a) identify the spatial progression patterns for DTBZ and RAC, which are expected to be different, and b) predict clinical metrics from the imaging data better than the mean tracer binding ratio (BR), thus demonstrating the clinical relevance of the captured patterns.

We first examine and compare the DTBZ and RAC binding patterns captured by PCA. The robustness and temporal evolution of the patterns are assessed by comparing them between the less- and more-affected sides of the brain, as well as between images obtained from subjects at different disease stages. We then combine several PCs using LASSO to construct cross-validated models of clinical disease metrics: disease duration (DD) and motor impairment scores. We compare the fit quality and prediction accuracy between the PC-LASSO models and the mean BR within an ROI. Finally, we analyze the distribution of voxel weights in the obtained PC-LASSO estimators in the context of PD pathology.

## Materials and methods

### Subjects and scans

The study included 10 healthy control (HC) subjects and 41 PD subjects combined from different clinical studies, but imaged using the same protocol on the same scanner. All except two PD subjects had both a DTBZ and a RAC scan; one PD subject lacked a DTBZ scan, and another lacked a RAC scan. Among the HC group, 6 subjects were scanned with both DTBZ and RAC, while another 4 subjects only had a DTBZ scan. Although the number of HC subjects was relatively low, their images were only used as part of a larger group that also included early PD subjects, as elaborated below. All HC and PD subjects underwent a T1-weighted structural magnetic resonance (MR) imaging scan.

Clinical and population characteristics of the HC and PD groups are listed in Table 1. To analyze the radiotracer binding patterns in different disease stages, the PD subjects were split into two sub-groups of approximately equal size according to the DD. The threshold that produced similarly-sized sub-groups was found to be 3 years. The sub-group with 0 ≤ DD ≤ 3 years (N = 22) is referred to as early PD, and the sub-group with DD ≥ 4 years (N = 19) is referred to as moderate PD. Since chronic intake of dopamine receptor agonist treatment, but not levodopa, has been shown to affect RAC binding [31], we recorded the agonist levodopa-equivalent dose (AgLED) for each PD subject.

**Table 1:** Population statistics.

	HC (RAC)	HC (DTBZ)	early PD (DD ≤ 3 years)	moderate PD (DD ≥ 4 years)
*N*	6	10	22	19
Sex	M = 3, F = 3	M = 7, F = 3	M = 13, F = 9	M = 12, F = 7
Age (years)	48.6 ± 17.9	48.4 ± 19.0	60.9 ± 9.3	62.6 ± 7.9
Disease duration (years)	na	na	2.1 ± 1.0	8.7 ± 3.8
H&Y ×*N*	na	na	1×3 1.5×1 2×18	1×4 1.5×1 2×11 2.5×1 3×2
Total MDS-UPDRS III	na	na	16.5 ± 8.3	22.7 ± 12.1
MoCA	na	na	27.8 ± 1.4	28.3 ± 1.3
Agonist status (mean AgLED)	na	na	OFF = 15 ON = 7 (155.4 ± 102.6)	OFF = 5 ON = 14 (177.6 ± 92.8)

The six HC subjects for which RAC images were acquired (first column) are included in the DTBZ HC group (second column). The age difference between the early PD and moderate PD groups was not statistically significant (p = 0.52). Likewise, there were no significant differences in the MoCA scores (p = 0.29) and the male-to-female ratio (p ≈ 1 according to the Fisher exact test). The calculation of the mean agonist levodopa-equivalent dose (AgLED, in mg/day) excluded subjects who were not on agonist therapy. H&Y = Hoehn and Yahr score; MoCA = Montreal cognitive assessment score.

The severity of movement impairment in the PD subjects was assessed off-medication (withdrawal period 12 hours) according to the motor subscale of the Movement Disorder Society’s Unified Parkinson’s Disease Rating Scale (MDS-UPDRS Part III) [32]. The better (less affected) and worse (more affected) sides were identified for each subject using the total MDS-UPDRS III scores for the left and right sides. As one of the clinical metrics for image-based prediction, lateral motor score (LMS) was computed by adding the individual items of MDS-UPDRS III for the leg (leg rigidity + toe tapping + leg agility) and arm (arm rigidity + finger tapping + hand movements + pronation/supination) assessments. The LMS was computed separately for the better and worse sides (Table 2). The tremor MDS-UPDRS III items were not included, as tremor is known to have worse correlation with dopaminergic deficit [33, 34]. The study was approved by the University of British Columbia Ethics Board and all subjects gave informed written consent.

**Table 2:** Distributions of the clinical metrics for all PD subjects.

Clinical metric	Min	Max	Mean	SD
LMS, better side	0	12	3.64	3.04
LMS, worse side	1	17	6.48	4.02
DD (years)	0	16	5.17	4.28

All PD subjects were included. LMS = lateral motor score; DD = disease duration; SD = standard deviation. Greater LMS corresponds to greater motor impairment.

### PET and MR image acquisition

PET scans of the PD subjects were performed with anti-Parkinson medication withdrawn at least 12 hours prior to scanning. Before tracer injection, transmission scans for attenuation correction were performed over 10 minutes with a rotating ^137^Cs source. Subsequently, the subjects were administered 320 ± 34 MBq of DTBZ and 322 ± 34 MBq of RAC via intravenous injection. PET data were acquired in list mode using a High Resolution Research Tomograph (HRRT, Siemens, spatial resolution (2.5 mm)^3^). Acquired coincidence data were binned into 16 temporal frames with frame durations of 1 min (×4 frames), 2 min (×3 frames), 5 min (×8 frames), 10 min (1 frame) (60 minutes in total). Dynamic images were reconstructed using the 3D list-mode ordinary Poisson ordered-subset expectation maximization (OP-OSEM) algorithm [35] with 16 subsets and 6 iterations, and corrections for attenuation, scattered and random coincidences. After reconstruction, the images were smoothed using a 2.0 mm (full width at half-maximum) Gaussian filter to reduce noise, and rigidly frame-to-frame realigned to correct for possible motion during the scans. The image dimensions were 256×256×207 with voxel size (1.219 mm)^3^.

MR images were acquired using a T1-weighted Turbo Field Echo sequence (TR 7.7 milliseconds) on a Philips Achieva 3T scanner. The acquired MR image dimensions were 256×256×170 with voxel size (1.0 mm)^3^. Post-acquisition, the MR images were resampled using trilinear interpolation to match the PET voxel size (yielding new dimensions 211×211×140 voxels). All subcortical structures included in the analysis were automatically segmented from the MR images using Freesurfer 6.0 [36].

Segmentations of the left and right putamen and caudate were used for the main part of the analysis. The ventral striatum (VS) regions, also segmented by Freesurfer (labeled as the accumbens area), are more closely related to the executive rather than motor function. Therefore, they were combined with the caudate segmentations to reduce the number of analyzed regions.

### Computation of parametric PET images

DTBZ and RAC images averaged over 30–60 min post-injection were rigidly co-registered to the corresponding subject’s MR images, using the SPM 12 software (www.fil.ion.ucl.ac.uk/spm/); the quality of the registration was visually inspected for each subject. Following co-registration, parametric BR images of DTBZ and RAC were computed for each subject, by dividing the voxel values in the respective activity images by the mean activity in a reference region. Freesurfer segmentation of the cerebellum (gray and white matter) was used to define the reference region for RAC. Segmentation of the occipital cortex, produced by masking the Freesurfer segmentation of the cortex, was used to define the reference region for DTBZ.

We used parametric images of BR rather than more commonly used BP_ND_, since the BR can be readily computed from static as well as dynamic scans. In a preliminary study, the proposed method was applied to parametric BR as well as BP_ND_ images, obtained using the simplified reference tissue model (SRTM2) [37, 38]; the results were found to be very similar. This was expected, since the BR (measured after the tracer equilibration) has been previously shown to be highly correlated with BP_ND_ values [39]. In addition, BR is often used as the outcome in imaging studies with AV-133, the ^18^F-labelled version of ^11^C-DTBZ [40]. In principle, PC-LASSO can be applied to different types of parametric images, since it primarily works with covariance patterns rather than absolute voxel values.

### Adjustment of disease duration

We found that for DTBZ, the mean BR in the putamen was better fitted against DD by an exponential function, rather than by a linear function, in agreement with previously reported results reported with BP_ND_ [41]. Thus, to enable the use of linear fits produced by LASSO, we linearized DD to obtain an adjusted DD (aDD) computed as *aDD* = exp(−0.17 × *DD*), followed by re-normalization between min(*DD*) = 0 years and max(*DD*) = 16 years. The coefficient −0.17 years^-1^ was found from the exponential fit of the mean DTBZ BR against DD. The re-normalization was applied to make aDD increase with disease progression, within the same range as DD. This facilitated the interpretation of the results (scatter plots and prediction errors).

### Processing and PCA of PET images

**Fig 1:**
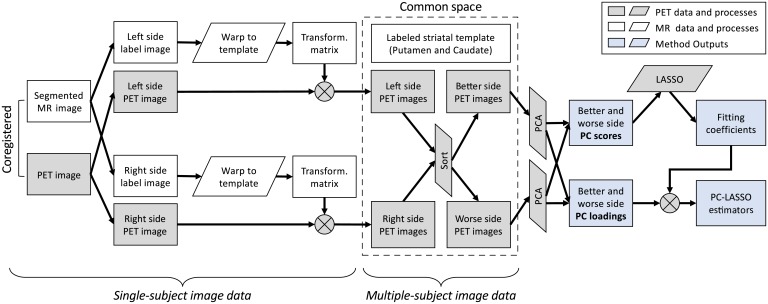
Schematic illustration of the image processing and analysis pipeline. Parametric PET images of the left and right striatum were warped to a common space using an MR-derived striatal template. In the common space, the images were re-sorted according to the better (less clinically affected) and worse sides, and used in PCA to obtain side-specific PC loadings and PC scores. The PC loadings and scores in the putamen and caudate were analyzed separately. LASSO was used to obtain the fitting coefficients between the PC scores and clinical PD metrics—aDD and LMS. Using the fitting coefficients, the PC loadings were linearly combined to obtain the PC-LASSO estimators.

**Fig 2:**
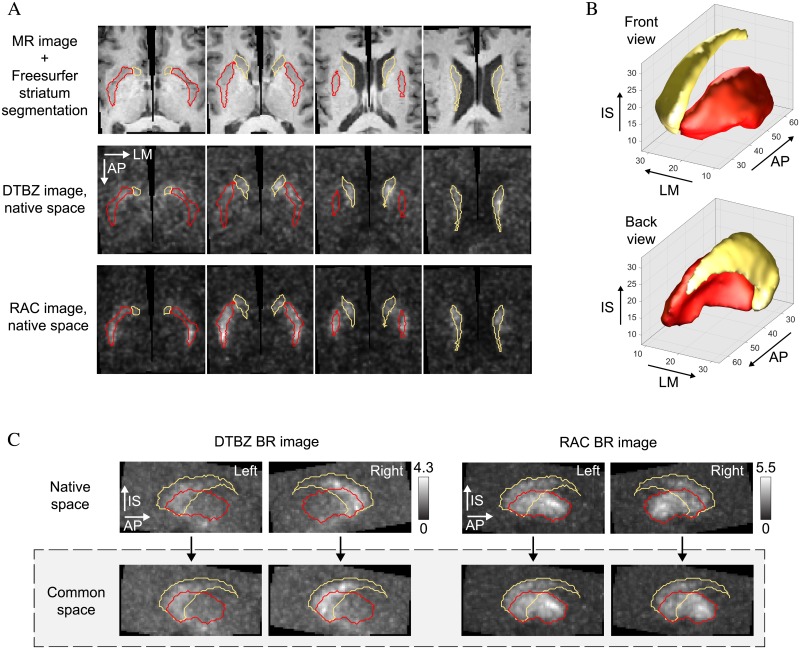
Examples of striatum images and segmentations in subject-native and common spaces. A: Segmented MR image, DTBZ and RAC images in the native space for a PD subject. B: Rendering of the striatal template from two views. To generate the template, Freesurfer segmentations of the MR images of the putamen and caudate in the HC subjects were rigidly co-registered and averaged (N = 10, one side chosen randomly from each subject). C: Comparison of DTBZ and RAC BR images in the subject-native and common spaces (PD subject, same as A). AP = anteroposterior, LM = lateromedial, IS = inferosuperior.

The pipeline for processing of DTBZ and RAC parametric BR images is schematically illustrated in Fig 1. For each subject, the segmented (and co-registered) MR and BR images were separated into left and right sides, and each side was processed separately. Examples of MR images, striatum segmentations, and PET images for one of the PD subjects are shown in Fig 2A. On each side, the MR image-defined segmentations of the putamen and caudate were combined to generate a single labeled volume that was warped to a common striatal template (Fig 2B) using 3D diffeomorphic mapping [42]. The resulting transformation was saved and applied to the respective BR image. Thus, the left and right striatal BR images of all HC and PD subjects were warped to a common labeled reference space (Fig 2C). The PD images were further sorted into better and worse brain sides, contralateral to the less and more clinically-affected body sides.

PCA was applied to the voxel BR data in the common space, separately for voxels in the putamen and caudate, and separately for the better and the worse brain sides. Each voxel in the common space was treated as a variable, and voxel BRs corresponding to different subjects were treated as observations. PCA finds orthogonal axes in the original variable space, termed PC loadings, that are aligned with the directions of greatest variance in the data. The following decomposition of the BR data into *K* PCs was performed:

$$\begin{array}{c} \vec{f_{n}}=\sum_{k=1}^{K}s_{nk}\vec{\omega_{k}} \end{array}$$ (1)

where $\vec{f_{n}}$ is the vector of voxel BR values of the *n*-th subject, $\vec{\omega_{k}}$ is the vector of voxel weights in the *k*-th PC, and *s*_*nk*_ are the PC scores, quantifying the projections of the original data onto the new basis $\vec{\omega_{k}}$. In other words, PC scores *s*_*nk*_ quantify the relative contribution of the *k*-th PC to the BR values of the *n*-th subject. Vectors $\vec{\omega_{k}}$ are the orthogonal PC loadings that define the contribution of each voxel to the PCA-defined basis functions.

### Spatial analysis of PC loadings

PC loadings obtained from PCA of all PD subjects were analyzed. The spatial patterns in voxel weights, captured by the PC loadings, were visually examined in the putamen and caudate. The better-side patterns were compared to the worse-side patterns. PC scores for each subject corresponding to these loadings/patterns were used in the multivariate PC-LASSO fits to obtain PC-LASSO models of aDD and LMS.

To assess the consistency of the patterns in different disease stages, PCA was additionally applied to the DTBZ and RAC caudate and putamen images of three subject sub-groups:

1. mixed HC and PD with DD ≤ 2 years (N = 11 PD + 6 HC for RAC, 12 PD + 10 HC for DTBZ)
2. early PD (DD ≤ 3 years, N = 22)
3. moderate PD (DD ≥ 4 years, N = 19)

The mixed HC/PD sub-group was constructed with the intention to capture the patterns of tracer binding associated with the onset of clinical disease. It consisted of the HC subjects, and a subset of subjects from the early PD sub-group (with DD up to two years). Only one brain side (left or right) was used from each HC subject, chosen randomly. The age difference between the HC and PD subjects in the sub-group was not statistically significant (p > 0.1). One PD subject in the mixed sub-group was on agonist therapy—that subject was excluded from the PCA of RAC images. The early and moderate PD sub-groups (Table 1) were intended to capture the patterns of tracer binding at later stages of the disease.

### PC-LASSO models for clinical metric prediction

Following the analysis of PC loadings, we used LASSO-based fitting with cross-validation to combine multiple PCs in a model optimized for prediction of clinical disease metrics. For a general set of input (predictor) and output (predicted) variables, LASSO finds a vector of fitting coefficients $\hat{\beta }$ such that

$$\begin{array}{c} \hat{\beta }=\underset{\beta }{\text{arg}\;\min}(\frac{1}{2}{\sum_{n=1}^{N}(y_{n}-\beta_{0}-\sum_{j=1}^{J}x_{nj}\beta_{j})}^{2}+\lambda \sum_{j=1}^{J}|\beta_{j}|) \end{array}$$ (2)

where *N* is the number of samples, *x*_*nj*_ are the input (independent) variables, *J* is the number of input variables, *y*_*n*_ is the outcome (dependent) variable, *b*_0_ is the intercept, and λ is a non-negative regularization parameter. Compared to the regular least squares fitting, regularization that partially restricts overfitting is achieved through the inclusion of the term $\lambda \sum_{j=1}^{J}|\beta_{j}|$. The choice of λ controls the degree of regularization. Large λ forces the terms with small *β*_*j*_ to go to zero. With λ → 0, LASSO becomes identical to regular multivariate least-squares fitting, and with λ → ∞ all but the constant (*β*_0_) terms are eliminated. LASSO is particularly well-suited for problems with a small sample size and a large number of independent variables.

The models that were fitted to the data using LASSO had the general form

$$\begin{array}{c} y_{n}=\beta_{0}+\sum_{j=1}^{J}s_{nj}\beta_{j} \end{array}$$ (3)

where the PC scores *s*_*nj*_ obtained from PCA of all available PD subjects are used instead of the variables *x*_*nj*_; *n* represents the subject index, and *j* represents the PC number. The PCs were numbered (ranked) in a descending order according to their variance-accounted-for (VAF). Based on the initial analysis of PC loadings, it was determined that large clusters of voxels with similar weights were only present in the top 5 PCs; loadings of PC6 and onwards resembled random noise. Thus, to make the number of pre-optimization independent variables constant in different tested models, only the scores of the top 5 PCs were used as LASSO inputs (*J* = 5).

The variable *y*_*n*_ represents the predicted clinical metric; we fitted separate models to predict aDD, better-side LMS and worse-side LMS (Table 2). With aDD, we tested better-side and worse-side PC scores as the input variables. In the models of better and worse LMS, PC scores from the corresponding contralateral side of the brain were used as inputs. All models with RAC data included age and AgLED as covariates. The total number (train+test) of samples in the cross-validated fitting was equal to 40 for both tracers.

Model optimization and fitting was performed using search over λ ranging from 0 to λ_*max*_ in 100 steps, where λ_*max*_ produced an intercept-only model (*β*_*j*_ ≡ 0). For each tested λ, the data were randomly divided 500 times into training and test sets using 0.3 holdout ratio: 70% of the data were used for training, and 30% for testing. The mean cross-validated mean squared error (MSE) in the test sets was measured (MSE_test_). For λ that produced the lowest mean MSE_test_ (λ_*min*_), MSE on the entire data was measured (MSE_all_). MSE_test_ was used to estimate the degree of overfitting and predictive accuracy for the unseen (future) data. MSE_all_ was used as a metric of model fit to the available data. Robustness of the LASSO fits corresponding to λ_*min*_ was assessed from the means and standard deviations of the term coefficients *β*_*j*_.

The MSEs of the PC-LASSO models of aDD and LMS given by Eq 3 were compared to those of 1) a linear model that only included the mean BR values, and 2) a constant model that included only the intercept *β*_0_. The constant model was included in the analysis to obtain reference MSE values that indicate a lack of meaningful outcome prediction. To assess the robustness of the PC-LASSO models, we analyze the LASSO trace plots of the fitting coefficients and MSE_test_ (i.e. their values as functions of λ). We compare the predicted-versus-actual (PVA) plots between the mean BR and PC-LASSO models, and provide the values and significance levels of the fitting coefficients *β*_*j*_ for aDD and LMS.

### Computation of PC-LASSO estimators

Since both PCA and LASSO are linear in nature, the LASSO-fitted combinations of PC scores that predict clinical metrics can be transformed back to the space of the original variables, i.e. voxels. Indeed, using the orthogonality of PCs and Eq 1, a LASSO-fitted linear model given by Eq 3 can be expressed as

$$\begin{array}{c} y_{n}=\beta_{0}+\sum_{j=1}^{J}\left(\vec{f_{n}}\cdot \vec{\omega_{j}}\right)\beta_{j} \end{array}$$ (4)

$$\begin{array}{c} =\beta_{0}+\left(\vec{f_{n}}\cdot \vec{v}\right) \end{array}$$ (5)

where the vector $\vec{v}=\sum_{j=1}^{J}\beta_{j}\vec{\omega_{j}}$ is termed the PC-LASSO estimator for the predicted clinical metric. PC-LASSO estimators represent weighted combinations of input voxels that optimally (i.e. with minimum cross-validation error) explain the outcome variables, e.g. clinical metrics of disease. Although the signs of PC loadings and scores are not uniquely determined in PCA, in PC-LASSO estimators the sign is determined in the process of model fitting. We visualize and interpret the spatial distribution of voxel weights in the PC-LASSO estimators of aDD and LMS, to gain new insight into the functional patterns associated with PD.

## Results

### Analysis of PC loadings computed using all PD subjects

**Fig 3:**
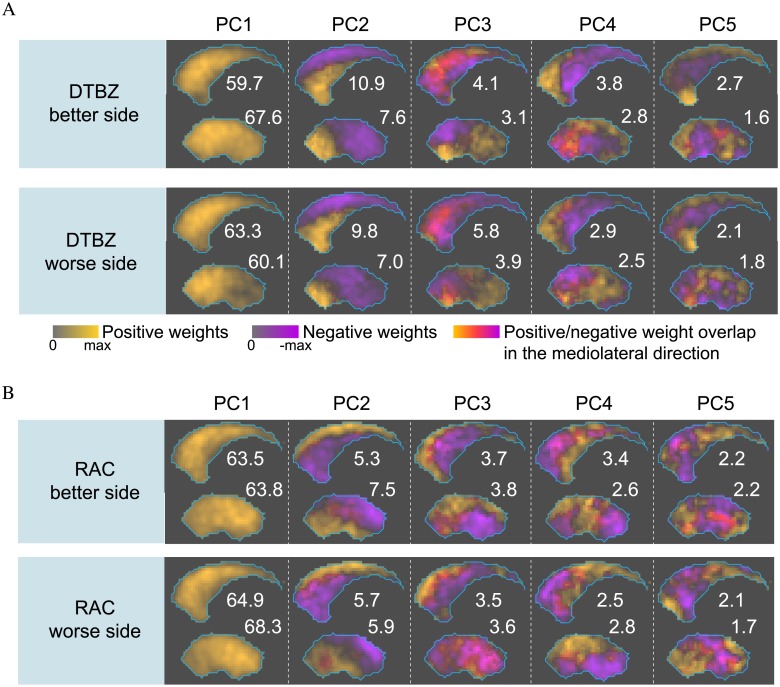
Maximum intensity projections of the better- and worse-side PC loadings, obtained from PCA of all PD subjects. Putamen and caudate loadings were computed separately, and ordered according to VAF expressed in percent. Positive and negative weights were projected separately and combined into a single composite image using different color scales. Color intensity reflects weight magnitude normalized to the maximum absolute value. Loadings with inverted color scales are equivalent. A: DTBZ PC loadings. B: RAC PC loadings.

DTBZ PC loadings in the putamen and caudate are visualized in Fig 3A in the order of decreasing VAF. Loadings of PCs with relatively high VAF contained large clusters of voxels with similar weights, while loadings with low VAF had more heterogeneous weight distributions. PC1 had much greater VAF compared to other PCs, and the respective loadings contained only positive voxel weights; thus, mathematically it represents a weighted mean, and describes a global variance in the tracer binding. PC2 loadings contained both positive and negative weights, and captured antero-posterior gradient in the putamen and infero-superior gradient in the caudate. Patterns reflected by PC3 loadings combined antero-posterior and infero-superior gradients. Patterns in the loadings of PC4 and PC5 were more intricate; PC5 in the caudate had prominent voxel weights in the ventral striatum region.

In the putamen, the difference between the better and worse side PC loadings (Fig 3A) reflected the disease asymmetry: the worse-side patterns can be seen as more progressed better-side patterns. For example, in the PC2 loadings, on the worse side the positive/negative weight boundary was shifted towards the anterior putamen (inferior caudate) compared to the better side. There were no similar side-to-side differences in the caudate PC loadings, possibly indicating that the asymmetry was not present or was less pronounced in the caudate.

Patterns captured by the RAC PC loadings were different from the DTBZ patterns, with the possible exception of PC1 (Fig 3B). For example, patterns captured by DTBZ PC2 and 3 in the putamen could not be found among the RAC PC loadings; the gradient direction in the RAC PC2 loadings was different compared to DTBZ. Also in contrast to DTBZ, the disease asymmetry was not clearly manifested in the RAC PC loadings on the better and worse sides.

### PC loadings computed in different disease stages

**Fig 4:**
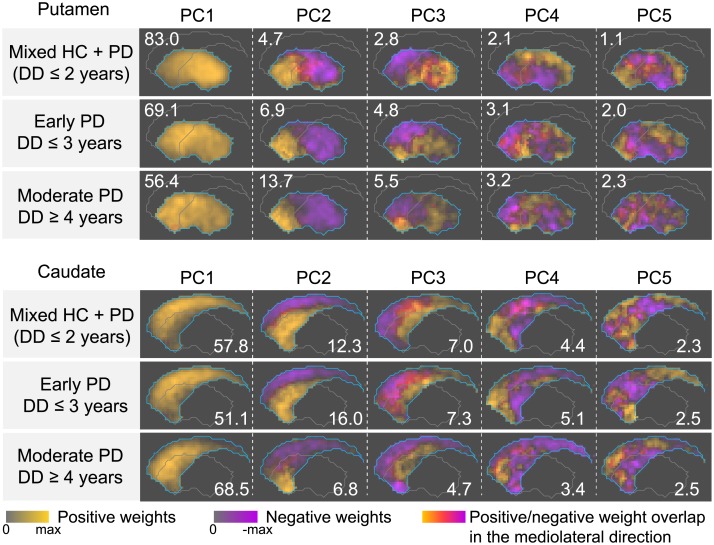
Maximum intensity projections of the better-side DTBZ PC loadings, obtained from PCA of subject sub-groups at different stages of the disease. The PCs are ordered according to VAF expressed in percent (shown in the corners). PC loadings in the putamen and caudate were computed separately. The first rows of the putamen and caudate loadings (mixed HC+PD) capture patterns associated with the initial onset of clinical PD symptoms. The second rows (early PD) represent stage of the disease shortly after symptom onset. The third rows (moderate PD) correspond to later stage of the disease.

Better-side DTBZ PC loadings computed in different subject sub-groups are visualized in Fig 4. The figure demonstrates that PCA produced generally similar PC loadings in different sub-groups of subjects, albeit with observable variations. In the putamen, VAF by the PC1’s in the sub-groups consistently decreased with disease progression, while VAF by PC2–5 increased. The voxel weight distributions in the loadings of PC1, 2 and 3 were found to progressively change as a function of disease stage. For example, in the mixed HC/PD sub-group, PC1 loading had greater weights in the posterior putamen (superior caudate), while in early and moderate PD, the region with higher weights shifted towards the anterior putamen (inferior caudate); in the putamen PC3 loadings, the ventral region became increasingly more involved, while the magnitude of the antero-posterior gradient decreased.

With RAC, voxel weight patterns in the PC loadings were also similar between different sub-groups (S1 Fig). However, in contrast to DTBZ we did not observe a gradual change in RAC PC loadings with disease progression.

### Performance and analysis of PC-LASSO models

The measured values of MSE_test_ and MSE_all_ for the DTBZ putamen models (constant, mean BR and PC-LASSO models) are summarized in Table 3. When putamen DTBZ data were used as the model inputs, PC-LASSO models of aDD and better-side LMS outperformed the mean BR and constant models. The largest improvement provided by PC-LASSO, compared to the mean BR, was in MSE_all_ on the worse side of putamen (mean BR MSE_all_ = 18.3, PC-LASSO MSE_all_ = 13.4). Although it is known that MSE_all_ has a tendency to diminish with greater number of independent variables, the fact that MSE_test_ of PC-LASSO was also lower implies that the optimized model was not overfitted. In the caudate, the MSE_test_ of all models were similar (Table 4).

**Table 3:** Prediction MSEs of DTBZ-based models (PUTAMEN).

Predicted metric	Input brain side	Input: Constant		Input: Mean BR		Inputs: PC1-5 scores (LASSO)		
		MSE_test_	MSE_all_	MSE_test_	MSE_all_	MSE_test_	MSE_all_	Retained terms
aDD	b.s.	21.6 ± 0.24	20.2	16.3 ± 0.17	14.3	14.4 ± 0.18	10.9	PC[1,3,4]
aDD	w.s.			20.3 ± 0.26	18.3	18.3 ± 0.29	13.4	PC[1,2,3,4,5]
b.s. LMS	x-lat.	9.8 ± 0.16	9.3	9.7 ± 0.15	8.7	8.9 ± 0.16	7.3	PC[1,2,4]
w.s. LMS	x-lat.	17.1 ± 0.28	16.1	18.8 ± 0.30	16.1	17.4 ± 0.27	16.1	none

The first two columns specify the predicted clinical metric (dependent variables) and the side of the brain from which the imaging data (independent variables) were taken. The last column specifies which terms were retained after optimization. The mean values and standard errors of MSE_test_ from 500 test sets are shown. A square root of the MSEs provides the absolute values of prediction errors. b.s. = better side; w.s. = worse side; x-lat. = contralateral.

**Table 4:** Prediction MSEs of DTBZ-based models (CAUDATE).

Predicted metric	Input brain side	Input: Constant		Input: Mean BR		Inputs: PC1-5 scores (LASSO)		
		MSE_test_	MSE_all_	MSE_test_	MSE_all_	MSE_test_	MSE_all_	Retained terms
aDD	b.s.	SAME AS PUT		18.0 ± 0.20	16.0	18.1 ± 0.19	16.0	PC[1]
aDD	w.s.			17.9 ± 0.23	16.1	17.5 ± 0.22	13.5	PC[1,2,5]
b.s. LMS	x-lat.			9.8 ± 0.15	8.8	9.8 ± 0.18	9.3	none
w.s. LMS	x-lat.			18.7 ± 0.30	16.1	17.1 ± 0.26	16.1	none

b.s. = better side; w.s. = worse side; x-lat. = contralateral.

When RAC data were used as the model inputs, the prediction errors were similar between the PC-LASSO, mean BR and constant models, indicating that very little aDD and LMS-related information, if any, is captured by this tracer in general.

We examined in detail three fitted DTBZ PC-LASSO models with lower MSE_test_ compared to that of the mean BR and constant models:

1. aDD predicted from the better-side PC 1, 3, and 4 scores in the putamen (scores that were reatined as inputs by LASSO)
2. aDD predicted from the worse-side PC 1–5 scores in the putamen
3. better-side LMS predicted from the contralateral PC 1, 2, and 4 scores in the putamen

**Fig 5:**
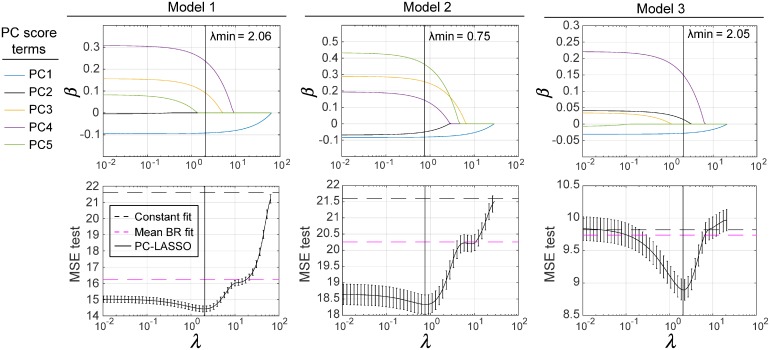
Trace plots of the coefficients *β*_*j*_ and cross-validated MSE_test_ for the best three PC-LASSO models. The coefficients *β*_*j*_ were fitted using the entire data. For comparison, the MSE_test_ produced by the mean BR and constant models are indicated by dashed horizontal lines. λ_*min*_ denotes the location of the global minimum in MSE_test_ (indicated by vertical lines).

These three models had the general form given by Eq 3 with different input variables, coeffients and predicted variables. Trace plots of the fit coefficients *β*_*j*_ and MSE_test_ against λ for the three considered DTBZ PC-LASSO models are shown in Fig 5. The MSE_test_ of the mean BR and constant models are plotted for reference. Distinct global minima were observed in the MSE_test_ of all three PC-LASSO models; these minima were located at non-zero values of λ, and were considerably lower than the MSE_test_ measured with the mean BR, PC1 scores alone, or constant models. With large values of λ, only the PC1 scores were retained.

**Fig 6:**
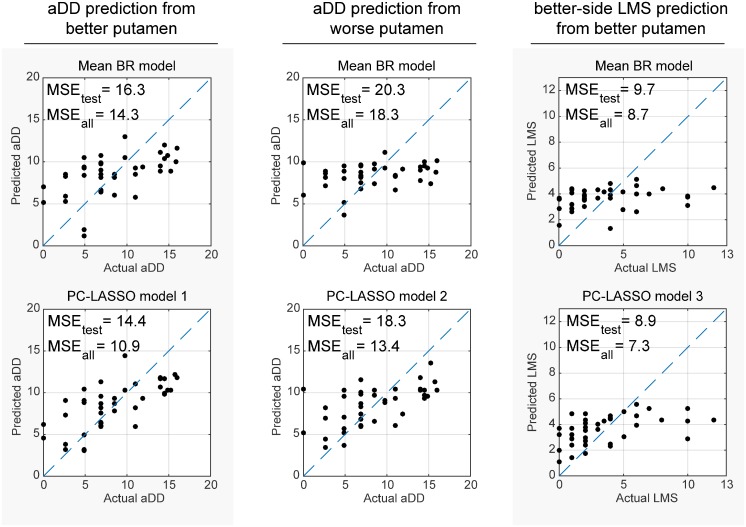
Predicted clinical metrics plotted against the actual metrics. The mean BR model predictions are shown on the top, and the PC-LASSO model predictions are shown on the bottom.

The PVA data plots for the PC-LASSO models and the respective mean DTBZ BR models are shown in Fig 6. The plots demonstrate that the PC-LASSO models indeed fit the data better than the mean BR. Improvement of the aDD fit with PC-LASSO was observed over the entire range of actual aDD values. Improvement of the LMS fit was primarily observed in the intermediate range (2–6) of LMS; the high actual LMS values (LMS > 6) were severely under-estimated.

**Fig 7:**
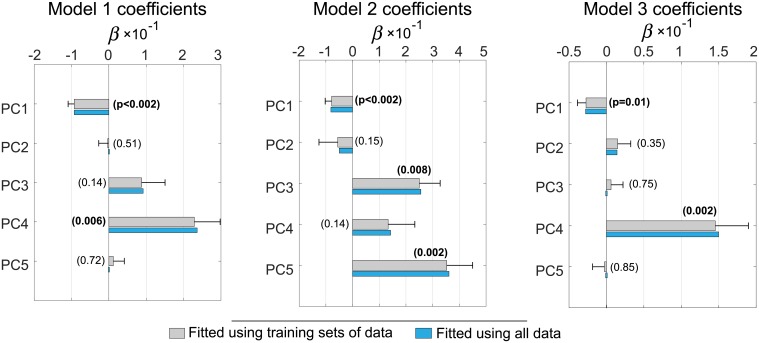
The mean values, standard deviations, and p-values of the fit coefficients *β*_*j*_. The means and standard deviations (error bars) were obtained from 500 training sets. The coefficients that were fitted on all available data are plotted for comparison. Values of p < 0.05 are highlighted with bold font.

The cross-validated mean values, standard deviations, and significance levels (p-values) of the fit coefficients *β*_*j*_, obtained with λ = λ_*min*_, are plotted in Fig 7. The mean values of the coefficients obtained from the training sets agree well with the values that were determined using the entire data. As indicated by the relative magnitudes of the means and standard deviations in Fig 7, the fit coefficients were consistent between different training sets. Interestingly, the contribution of PC4 in the model 3 was more significant than that of PC1. This may imply that the severity of motor impairment in PD is associated with neurodegeneration in specific sub-regions of the putamen, rather than in the entire structure.

### DTBZ PC-LASSO estimators of aDD and LMS

**Fig 8:**
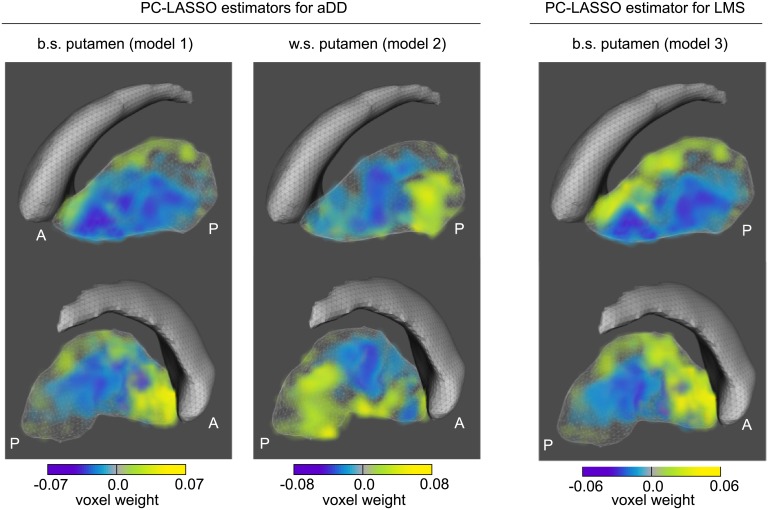
Voxel weight distributions in the DTBZ-based PC-LASSO estimators of aDD and better-side LMS in the putamen. Caudate is shown for spatial reference. The estimators were computed using *β*_*j*_ that were fitted on all data. Front view at the top, back view at the bottom. A = anterior; P = posterior.

The PC-LASSO estimators for aDD and LMS, corresponding to the three considered DTBZ models fitted on all data, are visualized in Fig 8. The better putamen estimator for aDD contained positive weights primarily in the anterior region, while negative weights were located in the interior and posterior regions. The worse putamen estimator for aDD featured negative weights in the anterior putamen, and positive weights in the posterior putamen—opposite to the corresponding better-side estimator for aDD. The estimator for the better-side LMS featured a positive-to-negative gradient in the ventral-to-dorsal direction.

## Discussion

### Robustness and clinical relevance of patterns captured by the PC loadings

One of the main findings obtained with PCA was that tracer binding patterns captured by the PC loadings were generally similar between different sub-groups of subjects. This provides evidence that the patterns were robust and inherent to the disease, and are thus very likely to be found in other PD cohorts. Comparison of DTBZ PC loadings between the sub-groups on a finer level uncovered the spatial evolution of patterns as a function of disease stage.

The pattern captured by PC1 describes a global, but not uniform, decrease in tracer binding. From the analysis of VAF it follows that in early disease, the majority of the variance between the subjects is explained by this pattern. The pattern of PC2 captured the antero-posterior gradient in DTBZ binding that develops in PD. The gradients captured by PC3, PC4 and PC5 describe variances of tracer binding in various directions that may be related to individual differences in disease progression. Although the gradient patterns of PC2 and higher order PCs reflected a much smaller component of variance compared to PC1, according to the VAF values they become more prominent with disease progression.

The difference between the better- and worse-side DTBZ PC loadings in the putamen was a reflection of the well-known asymmetrical nature of PD. Surprisingly, no corresponding side difference was found in the caudate loadings (Fig 3A). This suggests that the spatiotemporal progression of neurodegeneration may be symmetrical in the better and worse sides of the caudate, at least during the symptomatic stages of the disease.

Although it is very well known that pre- and post-synaptic processes respond differently to the disease, PCA was able to identify more accurately the spatial differences between the DTBZ and RAC binding alterations. Voxel-wise comparison of DTBZ and RAC second PCs in the putamen shows that pre- and post-synaptic functional gradients differ in terms of spatial localization. The predominant DTBZ gradient was in the antero-posterior direction, while the predominant RAC gradient was in the infero-superior direction (Fig 3).

In contrast to DTBZ, the binding patterns of RAC did not change with disease progression. These observations indicate that compensatory upregulation of the D2 receptor density may not exactly match the spatial localization of the dopaminergic terminal loss. The superior putamen is known to be primarily involved in the leg motor control [43]; it is thus possible to speculate that the limited spatial extent of the D2 receptor density upregulation, localized mostly in the superior putamen as captured by the putamen RAC PC2, is partially responsible for the fact that most new PD diagnoses are made based on the impairment of arm (but not leg) movement.

### Performance and biological interpretation of the PC-LASSO estimators

In terms of the clinical metric prediction error, PC-LASSO models provided an improvement over the mean BR models when using DTBZ imaging data, but not when using RAC. The fact that PC-LASSO did not always outperform the mean BR or constant models demonstrates that a greater number of independent variables did not automatically produce better prediction accuracy, and validates the method as being resistant to overfitting.

The DTBZ PC1 score appeared to be the strongest single predictor of aDD and LMS as it was the only term retained at higher values of λ. However, the final optimized models fitted using λ_*min*_ (with lowest MSE_test_ and MSE_all_) retained non-zero terms for PC2–5, i.e. terms that were different in meaning (and orthogonal) to PC1. This implies that the scores of PC2–5 had independent explanatory value while capturing clinically-relevant tracer distribution patterns.

Similarly to individual PC loadings, the obtained DTBZ PC-LASSO estimators for aDD and LMS (Fig 8) can be spatially interpreted. Taking into consideration the signs of the fit coefficients (Fig 7), it follows that on the better side of putamen, greater aDD is associated with increasing antero-posterior gradient, whereas on the worse side, it is associated with decreasing gradient. The latter is likely a reflection of the tracer binding floor effect in moderate-to-advanced disease.

Another observation of interest is that according to the putamen PC-LASSO estimator for the LMS, motor impairment in limbs is associated with the magnitude of the ventral-to-dorsal gradient in DTBZ binding. The found association between the ventral-to-dorsal gradient and limb motor scores appears to be consistent with the known striatal somatotopy in healthy state, wherein the forelimb and hindlimb cortical regions project to the ventral-to-dorsal putamen [43]. The lack of accurate PC-LASSO predictions for higher LMS values likely implies that DTBZ (and RAC) imaging data only provide a partial characterization of the neural circuitry that is responsible for motor function. Notwithstanding this limitation, the PC-LASSO estimators for aDD and LMS visually demonstrate how different properties of the same PET image (in this case gradients along different directions) may be related to different clinical metrics.

### Method limitations and future work

There are several limitations to the proposed method. First, LASSO was used to fit linear models, while the DTBZ binding is known to change non-linearly as a function of disease progression [44]. To overcome this limitation, we computed aDD for model fitting; however, the appropriate model for linearizing other disease metrics, or binding of other tracers, may not always be known a priori. Second, PCA does not uniquely determine the sign of $\vec{\omega_{k}}$ in Eq 1. This property may complicate identification of similar PC loadings obtained in different datasets. In future work, it would be beneficial to establish a metric of similarity between PC loadings that could account for possible sign differences, as well as small differences in voxel weights (e.g. between better and worse sides). We envision the use of some form of clustering to group similar PC loadings together. Third, the study was performed on cross-sectional data, which are not optimal when investigating disease progression. Other limitations of the method are the facts that it requires non-rigid image registration, and may only be applicable to tracers that have consistent/reproducible binding patterns.

Various modifications of the PC-LASSO pipeline can be envisioned. Our rationale for using PCA here was that, among similar techniques, it is most general, and is well-suited to capture intensity gradients through the inclusion of both positive and negative voxel weights. PCA loadings are orthogonal by construction, and the corresponding scores are expected to be uncorrelated, which makes LASSO appropriate to perform regularized fitting. However, depending on the application, PCA in principle can be replaced with another linear dimensionality reduction technique, such as sparse and/or generalized PCA [45, 46] or non-negative matrix factorization [47]. While the linearity of PCA did not pose an obstacle in our work, a non-linear dimensionality reduction method (e.g. kernel PCA) may also be used where it is required. In turn, LASSO can be replaced with logistic LASSO, group LASSO, elastic net, ridge regression, or a more sophisticated non-linear machine learning algorithm.

It would be of interest to compare the predictive accuracy of PC-LASSO models to that of frequently-used local radiomic features in different imaging scenarios; given that we only had a limited number of subjects available, such comparison was beyond the scope of this work. Future work could also include the use of PC-LASSO or similar method to predict disease progression in large longitudinal imaging study with multiple tracers. A larger number of subjects will also allow for a direct comparison of predictive performance between the PC-LASSO models and most frequently used radiomic features.

## Conclusion

We propose a novel data-driven method to construct models that predict clinical disease metrics from imaging data. The method is comprised of voxel-wise PCA followed by LASSO, and readily allows incorporation of clinical covariates at the same level as the voxel data. We applied PC-LASSO to the analysis of dopaminergic PET tracer binding in the striatum of PD subjects. The PC loadings obtained in different groups of subjects revealed predominant voxel-level binding patterns associated with the initial symptom onset and disease progression. The constructed DTBZ PC-LASSO models had lower cross-validated error of clinical metric prediction than the mean BR, confirming that patterns captured by the PC loadings are disease-related and offer additional explanatory and predictive power. The PC-LASSO estimators captured information in a non-local manner, and hence enabled data-driven visualization and interpretation of spatial patterns manifested in the images. The method is applicable to a variety of PET and SPECT imaging studies that focus on basal ganglia as well as other brain structures, including combination of data acquired with different tracers.

## Supporting information

S1 Fig Better-side RAC PC loadings at different stages of the disease. The PCs are ordered according to VAF expressed in percent (shown in the corners). PC loadings in the putamen and caudate were computed separately.
(EPS)

## References

[1] Eidelberg D. Metabolic brain networks in neurodegenerative disorders: a functional imaging approach. Trends in Neurosciences. 2009;32(10):548–557. 10.1016/j.tins.2009.06.003 19765835 PMC2782537

[2] Poston KL, Eidelberg D. FDG PET in the Evaluation of Parkinson’s Disease. PET clinics. 2010;5(1):55–64. 10.1016/j.cpet.2009.12.004 20689674 PMC2913894

[3] Niethammer M, Feigin A, Eidelberg D. Functional neuroimaging in Parkinson’s disease. Cold Spring Harbor Perspectives in Medicine. 2012;2(5):1–21. 10.1101/cshperspect.a009274 PMC3331691 22553499

[4] Gonzalez ME, Dinelle K, Vafai N, Heffernan N, McKenzie J, Appel-Cresswell S, et al Novel spatial analysis method for PET images using 3D moment invariants: Applications to Parkinson’s disease. Neuroimage. 2013;68:11–21. 10.1016/j.neuroimage.2012.11.055 23246861

[5] Martinez-Murcia FJ, Gorriz JM, Ramirez J, Moreno-Caballero M, Gomez-Rio M. Parametrization of textural patterns in 123I-ioflupane imaging for the automatic detection of Parkinsonism. Medical Physics. 2014;41(1):012502 10.1118/1.4845115 24387526

[6] Jain S, Salimpour Y, Younes L, Smith G, Mari Z, Sossi V, et al. Application of pattern recognition framework for quantification of Parkinson’s disease in DAT SPECT imaging. In: 2014 IEEE Nuclear Science Symposium and Medical Imaging Conference (NSS/MIC). vol. 7. IEEE; 2014. p. 1–8.

[7] Klyuzhin IS, Gonzalez M, Shahinfard E, Vafai N, Sossi V. Exploring the use of shape and texture descriptors of positron emission tomography tracer distribution in imaging studies of neurodegenerative disease. Journal of Cerebral Blood Flow and Metabolism. 2016;36(6):1122–34. 10.1177/0271678X15606718 26661171 PMC4908618

[8] Rahmim A, Huang P, Shenkov N, Fotouhi S, Davoodi-Bojd E, Lu L, et al Improved prediction of outcome in Parkinson’s disease using radiomics analysis of longitudinal DAT SPECT images. NeuroImage: Clinical. 2017;16(August):539–544. 10.1016/j.nicl.2017.08.021 29868437 PMC5984570

[9] Avanzo M, Stancanello J, El Naqa I. Beyond imaging: The promise of radiomics. Physica Medica. 2017;38:122–139. 10.1016/j.ejmp.2017.05.071 28595812

[10] Lambin P, Rios-velazquez E, Leijenaar R, Carvalho S, Granton P, Zegers CML, et al Radiomics: Extracting more information from medical images using advanced feature analysis. Proceedings of SPIE–the International Society for Optical Engineering. 2015;73(4):389–400.

[11] Yip SSF, Aerts HJWL. Applications and limitations of radiomics. Physics in Medicine and Biology. 2016;61(13):R150–R166. 10.1088/0031-9155/61/13/R150 27269645 PMC4927328

[12] Gillies RJ, Kinahan PE, Hricak H. Radiomics: Images Are More than Pictures, They Are Data. Radiology. 2016;278(2):563–577. 10.1148/radiol.2015151169 26579733 PMC4734157

[13] Parmar C, Grossmann P, Bussink J, Lambin P, Aerts HJWL. Machine Learning methods for Quantitative Radiomic Biomarkers. Scientific reports. 2015;5:13087 10.1038/srep13087 26278466 PMC4538374

[14] Hatt M, Tixier F, Pierce L, Kinahan PE, Le Rest CC, Visvikis D. Characterization of PET/CT images using texture analysis: the past, the present… any future? European Journal of Nuclear Medicine and Molecular Imaging. 2017;44(1):151–165. 10.1007/s00259-016-3427-0 27271051 PMC5283691

[15] Morris E, Chalkidou A, Hammers A, Peacock J, Summers J, Keevil S. Diagnostic accuracy of 18F amyloid PET tracers for the diagnosis of Alzheimer’s disease: a systematic review and meta-analysis. European Journal of Nuclear Medicine and Molecular Imaging. 2016;43(2):374–385. 10.1007/s00259-015-3228-x 26613792 PMC4700091

[16] Suwijn SR, van Boheemen CJM, de Haan RJ, Tissingh G, Booij J, de Bie RMA. The diagnostic accuracy of dopamine transporter SPECT imaging to detect nigrostriatal cell loss in patients with Parkinson’s disease or clinically uncertain parkinsonism: A systematic review. EJNMMI Research. 2015;5(1):1–8. 10.1186/s13550-015-0087-1 25853018 PMC4385258

[17] Pagano G, Niccolini F, Fusar-Poli P, Politis M. Serotonin transporter in Parkinson’s disease: A meta-analysis of positron emission tomography studies. Annals of Neurology. 2017;81(2):171–180. 10.1002/ana.24859 28019672

[18] Bailly C, Bodet-Milin C, Couespel S, Necib H, Kraeber-Bodéré F, Ansquer C, et al Revisiting the robustness of PET-based textural features in the context of multi-centric trials. PLoS ONE. 2016;11(7):1–16. 10.1371/journal.pone.0159984 PMC4965162 27467882

[19] Galavis PE, Hollensen C, Jallow N, Paliwal B, Jeraj R. Variability of textural features in FDG PET images due to different acquisition modes and reconstruction parameters. Acta Oncologica. 2010;49(7):1012–1016. 10.3109/0284186X.2010.498437 20831489 PMC4091820

[20] Blinder S, Klyuzhin I, Gonzalez M, Rahmim A, Sossi V. Texture and Shape Analysis on High and Low Spatial Resolution Emission Images. In: 2014 IEEE Nuclear Science Symposium and Medical Imaging Conference Record (NSS/MIC). Seattle, WA: IEEE; 2014.

[21] Klyuzhin IS, Gonzalez M, Shahinfard E, Vafai N, Sossi V. Exploring the use of shape and texture descriptors of positron emission tomography tracer distribution in imaging studies of neurodegenerative disease. Journal of Cerebral Blood Flow and Metabolism. 2016;36(6). 10.1177/0271678X15606718 26661171 PMC4908618

[22] Chalkidou A, O’Doherty MJ, Marsden PK. False discovery rates in PET and CT studies with texture features: A systematic review. PLoS ONE. 2015;10(5):1–18. 10.1371/journal.pone.0124165 PMC4418696 25938522

[23] Bzdok D. Classical Statistics and Statistical Learning in Imaging Neuroscience. Frontiers in Neuroscience. 2017;11(OCT). 10.3389/fnins.2017.00543 29056896 PMC5635056

[24] Bzdok D, Varoquaux G, Thirion B. Neuroimaging Research: From Null-Hypothesis Falsification to Out-of-Sample Generalization. Educational and Psychological Measurement. 2017;77(5):868–880. 10.1177/0013164416667982 29795936 PMC5965634

[25] Berk R, Brown L, Buja A, Zhang K, Zhao L. Valid post-selection inference. Annals of Statistics. 2013;41(2):802–837. 10.1214/12-AOS1077

[26] Bzdok D, Yeo BTT. Inference in the age of big data: Future perspectives on neuroscience. NeuroImage. 2017;155(April):549–564. 10.1016/j.neuroimage.2017.04.061 28456584

[27] Tibshirani R. Regression Selection and Shrinkage via the Lasso. Journal of the Royal Statistical Society B. 1996;58(1):267–288.

[28] de Lau LML, Breteler MMB. Epidemiology of Parkinson’s disease. The Lancet Neurology. 2006;5(6):525–35. 10.1016/S1474-4422(06)70471-9 16713924

[29] Dauer W, Przedborski S. Parkinson’s disease: mechanisms and models. Neuron. 2003;39(6):889–909. 10.1016/S0896-6273(03)00568-3 12971891

[30] Loane C, Politis M. Positron emission tomography neuroimaging in Parkinson’s disease. American journal of translational research. 2011;3(4):323–41. 21904653 PMC3158735

[31] Politis M, Wilson H, Wu K, Brooks DJ, Piccini P. Chronic exposure to dopamine agonists affects the integrity of striatal D2 receptors in Parkinson’s patients. NeuroImage: Clinical. 2017;16(July):455–460. 10.1016/j.nicl.2017.08.013 28879087 PMC5577411

[32] Goetz CG, Tilley BC, Shaftman SR, Stebbins GT, Fahn S, Martinez-Martin P, et al Movement Disorder Society-Sponsored Revision of the Unified Parkinson’s Disease Rating Scale (MDS-UPDRS): Scale presentation and clinimetric testing results. Movement Disorders. 2008;23(15):2129–2170. 10.1002/mds.22340 19025984

[33] Vingerhoets Francois JG, Schulzer M, Calne DB, Snow BJ. Which clinical sign of Parkinson’s disease best reflects the nigrostriatal lesion? Annals of Neurology. 1997;41(1):58–64. 10.1002/ana.410410111 9005866

[34] Doder M, Rabiner Ea, Turjanski N, Lees aJ, Brooks DJ. Tremor in Parkinson’s disease and serotonergic dysfunction. Neurology. 2003;60(4):601–605. 10.1212/01.WNL.0000031424.51127.2B 12601099

[35] Comtat, Bataille F, Michel C, Jones JP, Sibomana M, Janeiro L, et al. OSEM-3D Reconstruction Strategies for the ECAT HRRT. In: IEEE Symposium Conference Record Nuclear Science 2004.. vol. 6. IEEE; 2004. p. 3492–3496.

[36] Fischl B. FreeSurfer. Neuroimage. 2012;62(2):774–7781. 10.1016/j.neuroimage.2012.01.021 22248573 PMC3685476

[37] Wu Y, Carson RE. Noise Reduction in the Simplified Reference Tissue Model for Neuroreceptor Functional Imaging. Journal of Cerebral Blood Flow & Metabolism. 2002;22(12):1440–1452. 10.1097/00004647-200212000-00004 12468889

[38] Gunn RN, Lammertsma AA, Hume SP, Cunningham VJ. Parametric imaging of ligand-receptor binding in PET using a simplified reference region model. NeuroImage. 1997;6(4):279–87. 10.1006/nimg.1997.0303 9417971

[39] Lammertsma AA, Bench CJ, Hume SP, Osman S, Gunn K, Brooks DJ, et al Comparison of Methods for Analysis of Clinical [11C]Raclopride Studies. Journal of Cerebral Blood Flow & Metabolism. 1996;16(1):42–52. 10.1097/00004647-199601000-00005 8530554

[40] Villemagne VL, Okamura N, Pejoska S, Drago J, Mulligan RS, Chételat G, et al Differential diagnosis in Alzheimer’s disease and dementia with Lewy bodies via VMAT2 and amyloid imaging. Neurodegenerative Diseases. 2012;10(1-4):161–165. 10.1159/000334535 22261520

[41] Nandhagopal R, Kuramoto L, Schulzer M, Mak E, Cragg J, Lee CS, et al Longitudinal progression of sporadic Parkinson’s disease: a multi-tracer positron emission tomography study. Brain. 2009;132(Pt 11):2970–2979. 10.1093/brain/awp209 19690093

[42] Lombaert H, Grady L, Pennec X, Ayache N, Cheriet F. Spectral log-demons: Diffeomorphic image registration with very large deformations. International Journal of Computer Vision. 2014;107(3):254–271. 10.1007/s11263-013-0681-5

[43] Nambu A. Somatotopic Organization of the Primate Basal Ganglia. Frontiers in Neuroanatomy. 2011;5(April):1–9. 21541304 10.3389/fnana.2011.00026 PMC3082737

[44] de la Fuente-Fernández R, Schulzer M, Kuramoto L, Cragg J, Ramachandiran N, Au WL, et al Age-specific progression of nigrostriatal dysfunction in Parkinson’s disease. Annals of neurology. 2011;69(5):803–10. 10.1002/ana.22284 21246604

[45] Vidal R, Ma Y, Sastry S. Generalized principal component analysis (GPCA). IEEE Transactions on Pattern Analysis and Machine Intelligence. 2005;27(12):1945–1959. 10.1109/TPAMI.2005.244 16355661

[46] Zou H, Hastie T, Tibshirani R. Sparse principal component analysis. Journal of Computational and Graphical Statistics. 2006;15(2):265–286. 10.1198/106186006X113430

[47] Lee DD, Seung HS. Learning the parts of objects by non-negative matrix factorization. Nature. 1999;401(6755):788–791. 10.1038/44565 10548103

